# Simulating the origins of life: The dual role of RNA replicases as an obstacle to evolution

**DOI:** 10.1371/journal.pone.0180827

**Published:** 2017-07-10

**Authors:** Natalia Szostak, Jaroslaw Synak, Marcin Borowski, Szymon Wasik, Jacek Blazewicz

**Affiliations:** 1 Institute of Computing Science, Poznan University of Technology, Poznan, Poland; 2 Institute of Bioorganic Chemistry, Polish Academy of Sciences, Poznan, Poland; 3 European Centre for Bioinformatics and Genomics, Poznan, Poland; University of Ostrava, CZECH REPUBLIC

## Abstract

Despite years of study, it is still not clear how life emerged from inanimate matter and evolved into the complex forms that we observe today. One of the most recognized hypotheses for the origins of life, the RNA World hypothesis, assumes that life was sparked by prebiotic replicating RNA chains. In this paper, we address the problems caused by the interplay between hypothetical prebiotic RNA replicases and RNA parasitic species. We consider the coexistence of parasite RNAs and RNA replicases as well as the impact of parasites on the further evolution of replicases. For these purposes, we used multi-agent modeling techniques that allow for realistic assumptions regarding the movement and spatial interactions of modeled species. The general model used in this study is based on work by Takeuchi and Hogeweg. Our results confirm that the coexistence of parasite RNAs and replicases is possible in a spatially extended system, even if we take into consideration more realistic assumptions than Takeuchi and Hogeweg. However, we also showed that the presence of trade-off that takes into the account an RNA folding process could still pose a serious obstacle to the evolution of replication. We conclude that this might be a cause for one of the greatest transitions in life that took place early in evolution—the separation of the function between DNA templates and protein enzymes, with a central role for RNA species.

## 1 Introduction

The origins of life and their evolution are currently one of the hottest topics in science. Since the discovery of catalytic RNA activities [1, 2], the RNA World hypothesis has become the most plausible hypothesis for the origins of life [3]. Since its foundation [4–6], many researchers have performed experiments aimed at finding the most plausible pathways by which stereochemically appropriate nucleotides could build prebiotic RNAs. Recently, Powner et al. proved that the 2-aminooxazole pathway could yield pyrimidine nucleotides [7], while the formamide-based chemistry showed the possibility to create purines and pyrimidines in the presence of various catalysts [8]. Starting from the assumption that nucleotides were already present in the primordial soup, Costanzo et al. experimentally demonstrated RNA nucleotide self-polymerization [9] and Ferris et al. highlighted the role of mineral surfaces for increasing the length of the polymers [10].

Another problem related to the origins of life is that according to RNA World hypothesis, to spark life, the replication of existing RNA molecules would be necessary. RNA replication is not completely accurate; hence, as a consequence of mutation, evolution forms quasispecies that is the population of self-replicating polynucleotide chains [11–16]. As a result functional information can be easily lost in the so-called error catastrophe, especially due to the high mutation rates that probably characterized the first polymerases [17]. Despite this, researchers have performed many attempts to create the RNA polymerase ribozyme [18–22], recently resulting in a cross-chiral RNA polymerase ribozyme [23] and a system of cooperative RNA replicators [24], as well as RNA polymerase ribozyme that is able to synthesize structured functional RNAs, including aptamers and ribozymes [25]. However, these molecules are too large to be maintained in a quasispecies population, as they exceed the 100 nucleotide error threshold, which is the maximum length polynucleotide molecule that can be accurately replicated without high fidelity polymerases [13]. Eigen suggested hypercycles as a solution to the error threshold problem mentioned above [13, 26]. However, even if the traditional hypercycle model formulation based on ordinary differential equations (ODE) is ecologically stable, it is proved to be evolutionarily unstable [27]. In other words, hypercycles are not resistant to parasites defined as RNA molecules that do not have replicase catalytic activity but can be replicated by replicases [13, 28–34]. As the traditional hypercycle ODE model was homogeneous, the later works showed that the spatial consideration enhances the persistence of interacting individuals and allows for a stable coexistence of replicases and parasites [28, 30, 31, 33, 35–37]. Once the population structure is modeled, hypercyclic interactions are not necessary [38]. Moreover, Hogeweg and Takeuchi [32] showed by an *in silico* approach that the replicase-parasite system (RP system for short) is not only resistant to parasites, but more importantly, that parasites are responsible for the formation of the traveling wave patterns that ensured the evolutionary stability of the system.

As discussed above, many of the chemical and biological experiments that have been performed to date have focused on explaining abiogenesis. However, even though they have given us some answers and clues, it seems that the complexity of life makes it very tough for wet lab experimental analyses. Strikingly, mathematics and computing science have proven to be very useful for the analysis of many complex biological phenomena. Computational approaches are often faster and easier than performing wet lab experiments. Moreover, they allow for the careful tuning of a system’s parameters, which is often beyond the scope of traditional experiments [39]. Mathematical and computational approaches allow abstraction from the biochemical details of a system and enable researchers to focus on the core dynamics of a system that is responsible for the phenomena under consideration. This especially applies to studies on the origins of life and evolution, as we would like to test various scenarios with often limited knowledge about the details of their components. Moreover, *in silico* approaches are perfect for studying evolution because evolution often occurs on very large timescales that are hard to observe with standard methods. Nevertheless, when conducting *in silico* experiments, it should be kept in mind that the choice of a suitable modeling technique is crucial to ensure that the results give the correct answers for the problem under consideration.

There are many modeling techniques and simulation methods that can be used to analyze biological systems, including those that try to explain the origins of life. In this paper we focused on two modeling formalisms: cellular automata (CA) and multi-agent systems (MAS).

CA is represented as a grid of cells with a finite number of states that evolves in discrete-time [40, 41]. In the most general case, the state of a given cell at a given time depends only on its own state and the state of its neighbors at the previous time step. Various CA variants are currently one of the most actively studied methods for biophysical simulations [42]. CA utilizes a discrete representation of space and time.

More general approaches are multi-agent systems (MAS) that consist of a set of agents interacting in a given dynamic environment [43–45]. Agents are capable of autonomous actions and make decisions by taking into account their internal states and environments. A decision can modify the internal state of an agent, its behavior or its morphology, as well as the environment because the agent is able to act locally around it. MAS are well suited for modeling crowded environments because, similar to CA, they model global behavior through interactions between individual entities. Compared to the CA approach, the MAS approach is more suitable for modeling and simulating biological systems because it provides an easier way for representing the interactions between entities through agent interactions. Unfortunately, this modeling technique is not as well-supported by existing software as a CA, and simulating multi-agent systems usually requires the implementation of custom software. However, when implementing these approaches, authors can easily provide dedicated techniques, methods and tools for the modeling and simulation of biological systems [46, 47].

In this paper, we address the interplay problems between RNA replicases and RNA parasitic species in the origins of life context. This problem is important for understanding the pre-life events that led to current lifeforms and evolution that could govern the transition from the RNA world to the DNA-RNA-protein world. We asked whether parasites and replicases could coexist, what types of processes allowed this coexistence, and finally, if and how the coexistence between parasites and replicases could impact the further evolution of replicases themselves.

In our work, we aimed to verify and deepen the findings from Takeuchi and Hogeweg [33] regarding the replicase-parasite surface model. We also determined the implementation details of this model based on the analysis of source code received from Takeuchi. We believe that it can greatly help researchers who want to better understand this topic. As it has been previously shown that the methodology has a great impact on results and may therefore lead to varied interpretations [48], we propose to use multi-agent-based modeling to increase the precision of the model. We investigated the role of different attributes that characterize the agents and stability of the system consisting of interacting replicases and parasites, as well as its evolutionary dynamics. We also took a closer look at the ability of the system to evolve in a direction that promotes replication. Our results confirm the general findings of Takeuchi and Hogeweg [33] regarding multilevel selection. However, in a system with more realistic diffusion modeled with our agent-based methodology, more precise names for the observed phenomena could be *explosions of life* rather than *traveling waves*. The differences come from the continuous treatment of the space in the MAS model.

Moreover, we present results from our novel analysis, which show that even if stable coexistence is possible in a spatially extended system, this does not guarantee the evolution of better replicases. This happens because evolution tends to decrease the time replicases spend in a folded state, therefore increasing their availability as templates. As replication is performed by RNA replicases that also store the necessary information for creating new instances of themselves, the existence of a trade-off that takes into account RNA folding could still pose a serious obstacle to evolution. This dual nature of RNA replicases might itself be a cause for one of the greatest transitions in life that took place early during evolution—the separation of function between DNA templates and protein enzymes, with a central role for RNA species.

## 2 Materials and methods

The analysis of the replicase-parasite system (RP system) described in the 2.1 section required the design of several methods. The key method is the simulation algorithm. We based our multi-agent approach on the cellular automaton proposed by Takeuchi and Hogeweg [33]. Unfortunately, the above mentioned automaton was described without all the details required to reimplement it. To solve this problem, we asked authors for the algorithm source code and analyzed it to be sure that our approach is precisely equivalent of the original work, and so that we could credibly compare them. The detailed presentation of our algorithm is presented in the 2.2 section. The most difficult part of the algorithm was the conversion of the parameter values used by Takeuchi et al. in the multi-agent system. There are no commonly recognized general methods for converting parameter values between cellular automata and multi-agent systems, so we propose a new method described in the 2.6 section. Apart from this, the designed algorithm adapts some existing methods for simulating diffusion (section 2.3) and first and second-order reactions (sections 2.4 and 2.5 respectively).

### 2.1 Biological system

The model consists of a set of molecular species that interact in a two-dimensional toroidal environment that was chosen to avoid edge effects. Each molecule from each species is tracked individually in the simulation and has its own attributes stored in a state variable *x*_*i*_ for each molecule *i*. Starting from the initial distribution, the molecules are propagated through the space by an agent-based simulation. During each simulation step, the number of particles for each molecular species is recorded, as well as statistics related to the *l* and *a* attributes described below. The agent positions and attribute values are visualized during the simulation by creating a single image during each simulation step. To keep simulation computationally tractable, water molecules are not simulated. We based the simulation on mass action kinetics and modeled both first- and second-order reactions.

There are two singular molecule species simulated that are later denoted as uncomplexed; the parasites are denoted by P and the replicases are denoted by R. The replicases model the RNA chains with catalytic activities that are called ribozymes, as they are capable of performing RNA chain replication. The parasites model RNA chains that lack catalyzing replication abilities, but can be copied by the replicase species and are therefore considered to be parasite molecules in the modeled system.

During the simulation, uncomplexed molecules, replicases and parasites are characterized by two attributes that range from 0 to 1. The former is the agent affinity towards replicases, which is denoted by *a*, and models how well the molecule is recognized by replicases and its ability to form a stable complex with replicases. The second attribute is the probability of an agent being in a folded state, which is denoted by *l*. Agents can serve as templates for replicases in the unfolded state. In the folded state, replicases serve as catalysts, whereas parasites in this state are considered to be inactive in this study. During replication, the affinity towards replicases and the probability of being in a folded state can be slightly changed to mimic mutation events known from nature.

**Fig 1:**
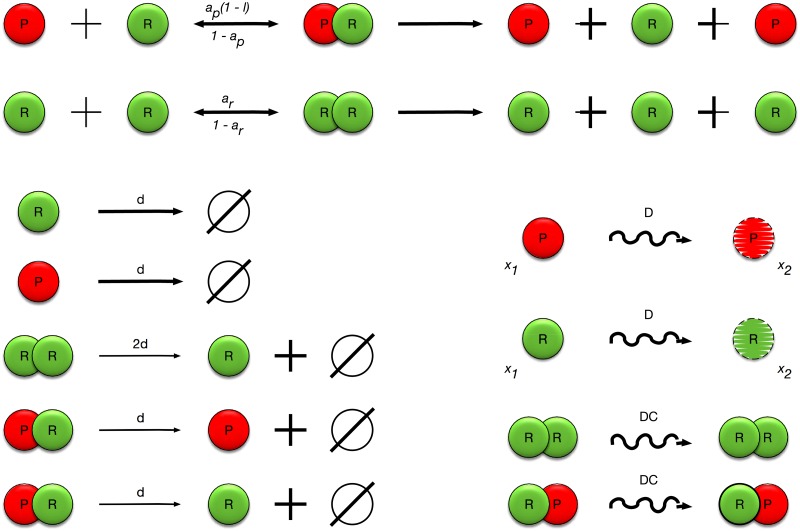
Interactions modeled by the multi-agent system. Parasites or replicases can form complexes with other replicases at *a*_*P*_(1 − *l*_*P*_) and *a*_*R*_(1 − *l*_*R*_) rates, respectively. A two replicase complex can either dissociate at a rate of (1 − *a*_*R*_) or produce a new replicase at rate *K*. Analogously, a replicase-parasite complex can either dissociate at a rate of (1 − *a*_*P*_) or produce a new parasite at rate *K*. Parasites and replicases can also decay at rate *d* and move at rate *D*. Similarly, complexes can move at rate *D*′. The parameters (rate constants) used in this simulation were following: *d* = 0.182; *D* = 0.75; *D*′ = 0.0476; *K* = 239800; Δ*t* = 1; ∀_*i*_ *r*_*i*_ = 0.5.

Two replicases can form a complex of two replicases and a replicase and a parasite can form a replicase-parasite complex. Each uncomplexed molecule is represented by an agent at a corresponding position and inherits its general properties from the molecular species in an object-oriented way. The agents are represented as circles of a given size. Complexes are virtual species that store information from the molecules that built them and are represented as two overlapping circles. The existence of a complex models the fact that the bimolecular reaction responsible replication occurs during the presence of a complex that uses its components as reactants. All of the interactions modeled by the system are presented in the Fig 1.

### 2.2 Simulation algorithm

The multi-agent simulation algorithm used to simulate the RP system is presented below. Each replicase and parasite is represented by a single agent in the simulation. When the agent is created, its remaining life time (RLT) is initialized random (see Eq 2), and during each simulation iteration, the time is decreased. When the complex is dissociated, the time does not have to be recalculated because of memoryless properties (see Section 2.4). There are a maximal number of allowed neighbors for each agent. If this is exceeded, the agent is removed due to a lack of resources. During each replication event, there is a certain probability of a mutation to agent properties with regard to agent affinity towards attributes or the probability of being in a folded state. The detailed algorithm is presented in the below list 1.

**Algorithm 1** Our algorithm.

1: Initialize the simulation.

2:  Initialize the number and positions of the agents from a file or randomly.

3:  Initialize all replicases with equal *a*_*R*_ and *l*_*R*_ values.

4:  Initialize all parasites with equal *a*_*P*_ and *l*_*P*_ values.

5: **while** ((simulation time < time limit) AND (there are both parasites and replicases (complexed or uncomplexed) present)) **do**

6:  Increase the simulation time by Δ*t*.

7:  Decrease the *RLT* time for each agent (complexes and uncomplexed).

8:  **for all** uncomplexed agents with the RLT = 0 **do**

9:   Remove the agents.

10:  **end for**

11:  Randomize the order of all agents.

12:  **for all** agent *x*_*i*_ **do**

13:   **if** *x*_*i*_ is complex AND *RLT*(*x*_*i*_) = 0 **then**

14:    Dissociate agents into two uncomplexed agents with the same position as the complex.

15:   **end if**

16:   **for all** agent that overlaps *x*_*i*_ (see Eq 3) **do**

17:    Add it to the set of neighbors *N*

18:   **end for**

19:   **if** |*N*|>*N*_*max*_ **then**

20:    remove agent *x*_*i*_ from the simulation

21:   **end if**

22:   Randomize the order of *N*.

23:   Move *x*_*i*_ (see Eq 1).

24:   **if** *x*_*i*_ is complex **then**

25:    Decrease the replication time (TTR).

26:    **if** *TTR* = 0 **then**

27:     Create new agent *x*′ equal to the template from complex *x*_*i*_.

28:     Mutate *x*′.

29:     Dissociate *x*_*i*_:

30:      Create agents *t* and *r* based on the complexed template and replicase.

31:      Copy the position of *x*_*i*_ to *t* and *r*.

32:      Restore the *RLT*(*r*) and *RLT*(*t*) values from the moment when the complex was created.

33:      Remove *x*_*i*_ from the simulation.

34:    **end if**

35:   **else**

36:    **for all** *n*_*j*_ ∈ *N* **do**

37:     **if** (*n*_*j*_ is uncomplexed) AND (*n*_*j*_ OR *x*_*i*_ is replicase) AND CheckReactionProbability(see Eq 4) **then**

38:      Create complex *x*′.

39:      Randomly initialize *TTR*(*x*′) (see Eq 2).

40:      Randomly initialize *RLT*(*x*′) (see Eq 2).

41:      Initialize the position of *x*′ as the average position of *n*_*j*_ and *x*_*i*_.

42:      Remove *n*_*j*_ and *x*_*i*_ from the simulation.

43:     **end if**

44:    **end for**

45:   **end if**

46:  **end for**

47: **end while**

### 2.3 Diffusion

Agents move in discrete time (Δ*t*) using continuous-time random walk to simulate the Brownian motion of molecules suspended in a fluid. The position of a molecule after each step of the simulation is calculated using the following formula [49]:

$$\begin{array}{c} \vec{x}\left(t+\Delta t\right)=\vec{x}\left(t\right)+\sqrt{2D\Delta t}\vec{\xi } \end{array}$$ (1)

where *D* is the diffusion coefficient and $\vec{\xi }$ is a Gaussian random two-dimensional vector with mean 0 and variance 1.

### 2.4 Unimolecular reactions

In the simulation, the following three types of unimolecular, first-order reactions are allowed: (1) decay, (2) replication and (3) dissociation. We assume that the state of the agent (in particular its age) does not determine the probability of a reaction. Moreover, the probability of a reaction does not depend directly on time. Hence, all first-order reactions are memoryless and the distribution of time periods after which reactions occur is a random variable with exponential distribution. To keep the simulation cost and time reasonable, instead of checking the probability of each reaction at each time step, it is possible to calculate the time of a given unimolecular reaction when a molecule is created using the following formula [50]:

$$\begin{array}{c} t_{action}=-ln\left(X\right)/k \end{array}$$ (2)

where *X* denotes a random number uniformly distributed in the range (0; 1] and *k* denotes the exponential distribution rate. Using this formula, we can simply simulate the reaction after *t*_*action*_ time instead of checking the probability of the reaction after each simulation step, as a mechanism for reducing the computational complexity. We use an exponential distribution because it is a good approximation of processes occurring in real systems.

To handle a decay process, we calculate the lifetime of each molecule by substituting the decay coefficient *d* for *k* in Eq 2. This is executed at the beginning of the simulation and then every time a new molecule is created during replication. We implemented a simulation consistent with the Takeuchi and Hogeweg model [33], where decay is allowed for both complexed and free agents. In this variant, when one of the complexed agents decays, the other agent is set free and no longer assumed to be in complex.

Replication and dissociation processes are also modeled as unimolecular reactions and the simulation algorithm handles them by calculating replication and dissociation times each time a complex forms between two molecules. For a replication, we substitute a coefficient of replication *K* for *k* in Eq 2. For a dissociation, we substitute a dissociation coefficient (1 − *a*) for *k* in Eq 2, where *a* is the affinity towards replicases by the agent performing the template role (see also section 2.5). During each replication event, there is a certain probability *μ*_*a*_ of a mutation on an agent’s affinity towards replicases. Moreover, after replication, complex is immediately dissociated, like in the case of bacteriophage T7 RNA polymerase [51]. After a complex dissociation, a new lifetime is assigned for each of the dissociated components.

### 2.5 Second-order reactions

In the simulation, two similar types of second-order reactions occur, the formation of a complex between a replicase and a parasite or between two replicases. As second-order reactions are dependent on the relative positions of the reactants, two molecules can react if they are within the reaction volume [52, 53], i.e., before the reaction can occur, agents have to move close to one another. In the simulation, we use the following collision distance:

$$\begin{array}{c} r_{c}=r_{i}+r_{j} \end{array}$$ (3)

as the maximum distance where two molecules can be to make reaction possible. Here, *r*_*i*_ and *r*_*j*_ are the radii of the circles representing the agents. In practice, for simplification, we assume that all agents have an equal size. For a specific agent, all of the agents that are in the reaction radius form the set of its neighbors. If the number of neighbors is greater than the maximum number of neighbors allowed, then the agent is erased. This is done to assure the proper load of an environment.

In reality, some collisions are elastic and do not trigger a reaction. This can be handled by introducing a reaction probability *ω*. To keep the simulation computationally reasonable, as we keep track of millions of molecules in each simulation, we adopted a simple strategy from Takeuchi and Hogeweg [33]. The reaction probability in each step is calculated by taking into account two template parameters, the affinity towards replicases, *a*, and the probability of being in a folded state, *l*. If one of the agents is a parasite, then it is selected as a template; otherwise, the replicase that did not initiate the reaction (denoted as *n*_*i*_ in the algorithm 1) is selected as the template. The probability is calculated with the following equation:

$$\begin{array}{c} \omega =a(1-l) \end{array}$$ (4)

Therefore, a reaction between two molecules happens if they are closer than a collision distance *r*_*c*_ and the random number *X*, which is uniformly distributed within the range [0, 1], is smaller than *ω*. If more than one agent is within the collision distance of the agent under the consideration, these agents are checked in random order until an agent that reacts is found or all the agents are processed. If none of the overlapping agents react, nothing happens, meaning that all the possible reactions were elastic. During each replication event, there is a certain probability *μ*_*l*_ of a mutation to the agent’s probability of being in a folded state.

### 2.6 Parameters

The choice of values for parameters is crucial for the behavior of the system. The current model is based on the Takeuchi and Hogeweg [33] CA model for parasites and replicases, and we wanted to keep the MAS model as similar as possible to allow for comparison between the two models. This is why we made an effort to adjust the values used in the Takeuchi and Hogeweg simulations to the MAS model. This was not strictly required, as both models use qualitative, rather than quantitative analysis. However, we preferred to keep their behavior as similar as possible.

For diffusion rate, it was possible to adjust its value analytically. However, others had to be adjusted experimentally by performing test simulations. The reason for this approach is that the approximation method strongly depends on the spatial distribution of agents, which makes simple conversion nearly impossible. In the following equations, parameters with a *CA* subscript represent parameters in Takeuchi’s cellular automaton model, while parameters without this subscript represent corresponding parameters in our agent-based approach.

The most elementary simulation parameter is the time step length Δ*t*. It determines the time scale that is used by the other parameters. Taking this into account, Δ*t* can have an arbitrary value. In our simulation, we set it to 1 unit because it is straightforward to use the value Δ*t* = 1 in equations. The method for computing diffusion is presented below.

#### 2.6.1 Diffusion

**Single agent** To compute the diffusion constant of an uncomplexed agent, we only consider actions that can occur during the lifetime of this particular agent during cellular automaton. This excludes decay and formation of a complex because both of them cause the end of the presence of agents either explicitly (decay) or implicitly (complex formation). Under these assumptions, the only action that can be applied to an uncomplexed molecule is diffusion. Because it is the only possible action, the probability that it will occur is equal to:

$$\begin{array}{c} p_{D_{CA}}=1 \end{array}$$ (5)

To compare the diffusion probability in Takeuchi’s approach with the diffusion constant in our multi-agent approach, the average squared lengths of the movement vectors (their variance) must be computed and weighted, i.e., multiplied by the probability defined above. When using a Moore neighborhood in the cellular automaton approach, a molecule can move to eight different cells. Half of them are adjacent with a distance equal to 1 and the remaining are diagonal with a distance equal to $\sqrt{2}$. The variance of movement vectors can be characterized by the following equation:

$$\begin{array}{c} \sigma_{CA}^{2}=\frac{1}{8}(4\cdot 1^{2}+4\cdot {\sqrt{2}}^{2})\cdot p_{D_{CA}}=\frac{3}{2}p_{D_{CA}} \end{array}$$ (6)

In a multi-agent simulation, the variance of the movement vectors can be computed based on the following equation:

$$\begin{array}{c} \sigma^{2}=2D\Delta t \end{array}$$ (7)

where *D* denotes the diffusion coefficient of an agent used at the multi-agent approach.

To assure that both simulations behave similarly, the following condition must be fulfilled:

$$\begin{array}{c} \sigma_{CA}^{2}=\sigma^{2} \end{array}$$ (8)

By substituting the following Eqs 6 and 7 we get:

$$\begin{array}{c} \frac{3}{2}p_{D_{CA}}=2D\Delta t \end{array}$$ (9)

$$\begin{array}{c} D=\frac{3p_{D_{CA}}}{4\Delta t} \end{array}$$ (10)

**Complex** In Takeuchi’s approach, a complexed molecule moves with two times lower probability than an uncomplexed one. As in the previous subsection, we have to consider actions that can occur during the lifetime of the complex. Similar to an uncomplexed agent, we omit the decay of complexed molecules and complex dissociation. However, in addition to diffusion, we also have to consider replication. That is why the probability of diffusion by an arbitrarily chosen complexed molecule is equal to following equation:

$$\begin{array}{c} p_{D_{CA}^{\prime }}=\frac{\frac{D_{CA}}{2}}{\frac{D_{CA}}{2}+K_{CA}} \end{array}$$ (11)

where *D*_*CA*_ denotes the diffusion rate and *K*_*CA*_ denotes the replication rate used at a cellular automaton approach.

Moreover, because each complex consists of two neighboring cells, when calculating the variance of the movement vectors, we have to take into account the movement of the center of a mass of the complex. When performing diffusion of the complexed molecule, we choose one of its 8 neighboring cells (in exactly the same way as for an uncomplexed molecule). The complex is moved to this cell, and the other molecule in the complex is moved to the emptied cell. One of these eight neighbors is the other molecule in the complex. Choosing this cell causes the exchange of only two complexed molecules, so the center of mass does not move. Choosing any of the remaining 7 cells causes the movement of the center of mass; in four cases the distance of movement is equal to $\sqrt{2}/2$, in one case it is equal to 1 and in two cases its equal to $\sqrt{10}/2$. Taking this into account, we can compute the variance of movement vectors with the following equation:

$$\begin{array}{c} \sigma_{C_{CA}}^{2}=\frac{1}{8}\left(4\cdot {\left(\frac{\sqrt{2}}{2}\right)}^{2}+1^{2}+2\cdot {\left(\frac{\sqrt{10}}{2}\right)}^{2}\right)\cdot \left(2p_{D_{CA}^{\prime }}\right)=2p_{D_{CA}^{\prime }} \end{array}$$ (12)

where $p_{D_{CA}^{\prime }}$ has to be multiplied by 2 because the complex consists of two cells and any of them can be chosen to initiate complex movement.

In the multi-agent simulation, the variance of movement vectors for a complex, just as for a single agent, can be computed based on the following equation:

$$\begin{array}{c} \sigma_{C}^{2}=2D^{\prime }\Delta t \end{array}$$ (13)

where D’ denotes a diffusion coefficient of a complex used at multi-agent approach.

To assure that simulations behave in a similar way, the following condition must be fulfilled:

$$\begin{array}{c} \sigma_{C}^{2}=\sigma_{C_{CA}}^{2} \end{array}$$ (14)

By substituting Eqs 12 and 13 we obtain the following final equation:

$$\begin{array}{c} 2D^{\prime }\Delta t=2p_{D_{CA}^{\prime }} \end{array}$$ (15)

$$\begin{array}{c} D^{\prime }=\frac{p_{D_{CA}^{\prime }}}{\Delta t} \end{array}$$ (16)

#### 2.6.2 Replication rate

In Takuechi’s model, in each time step, a complex can move, dissociate, decay (each of its parts separately) or replicate. Replication, similarly to decay, is performed with exponential distribution of waiting time, provided the availability of empty space is constant. In our multi-agent approach, diffusion is a separate step and replication or dissociation occurs during the time for a specific event. These actions are completely independent. Moreover, the replication itself is a multi-step process, so modeling it with Dirac Delta function would be more realistic. This trick forces the deterministic treatment of a probability density. In practice, the replication time is computed using an exponential distribution, similarly to other periods, where the variable *K* is the distribution rate parameter. Such method correctly models Dirac Delta function, because exponential distribution tends to Dirac Delta with the rate parameter tending to infinity. Hence, we had to set *K* to be extremely large, meaning that the time to replication can be achieved immediately after complex formation. However, this immediate replication does not mean that it always happens because the complex could dissociate earlier. If dissociation does not happen, then replication occurs.

## 3 Results

**Fig 2:**
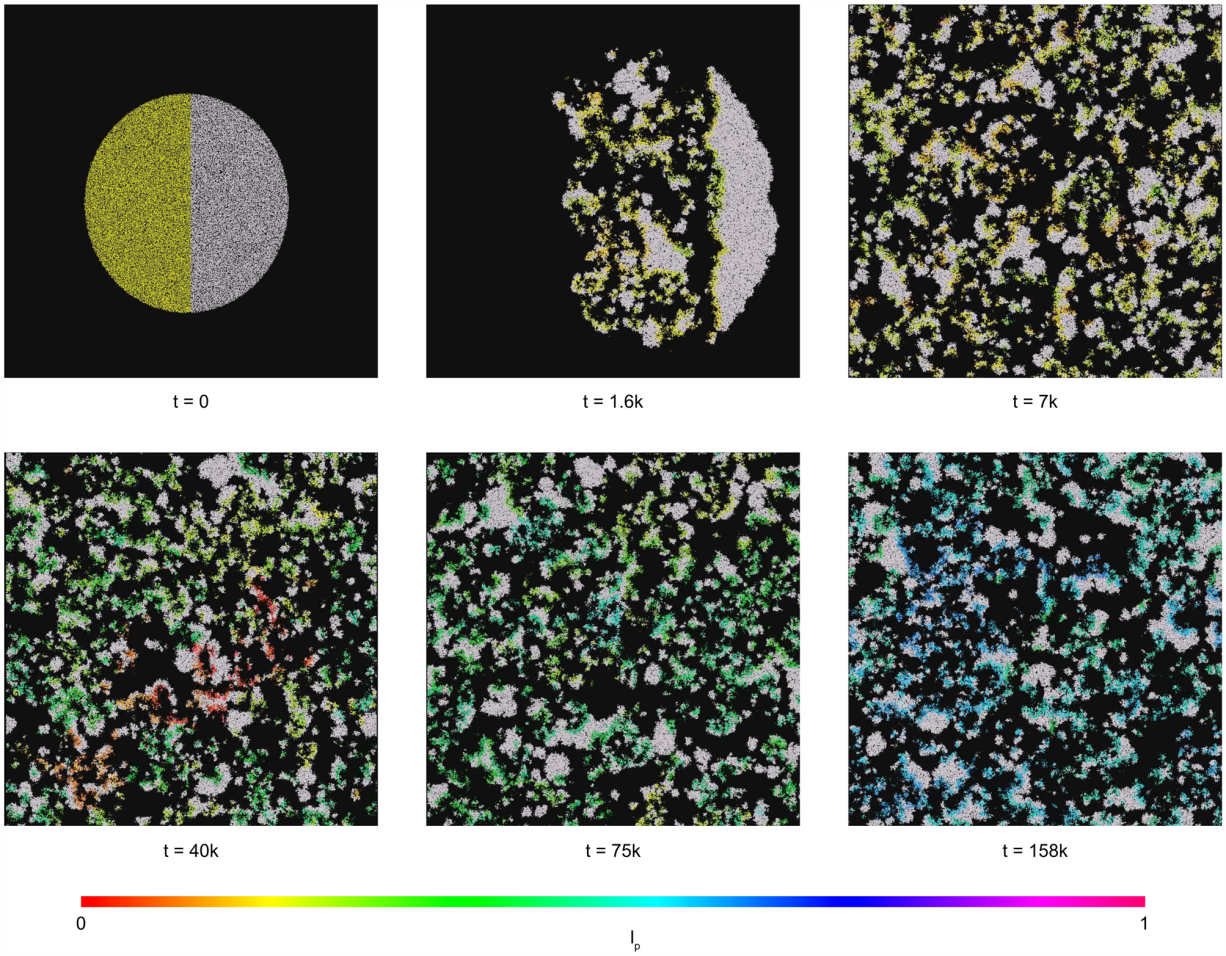
Images taken during the simulation of the first experiment. The presented frames were taken after 7k, 40k, 75k and 158k steps. The full movie is available as supplemental material S1 Video, S2 Video, S3 Video, and S4 Video. It is split into four parts because of file size constraints. It is also available in higher quality on the YouTube (https://www.youtube.com/watch?v=mKpiUH0iDoQ). The values of parameters used during the experiment are presented in Table 1 and label of Fig 1.

We simulated several variants with the multi-agent simulation described in the 2.2 Section. We always initialized the system with small replicase and parasite populations, both of which were of an equal size. We examined the random and nonrandom distribution of agents on a plane. In the latter case, the agents of one type were placed in a half of the circle, while the other type in the other half of the circle (Fig 2). We analyzed the case when only parasites were allowed to mutate, reproducing the experiment of Takeuchi and Hogeweg [33], and the case when both replicases and parasites could change their parameters. Each time, the parameter values *a* or *l* is presented on a chart; these values are the averages of these values from the whole simulated population in each simulation step. We assumed that the system will not go extinct if after stabilization parasites and replicases that form the system still coexisted. Moreover, we assumed that the stabilization occurred when the average value of the parameters stopped changing and the groups of agents were distributed uniformly in the space. This usually required no more than 150k simulation steps.

During experiments, we observed emergent behavior similar to the traveling waves described by Takeuchi and Hogeweg [33] in their CA simulations. Such waves consisted of groups of parasite and replicase agents changing their positions in constant directions similar to waves in water. The back side of the wave is formed by parasites that exploit replicases that constitute the front of the wave. As a result, parasites leave an empty space behind them, while new replicases are formed on the other side of the wave. This local extinction of parasites and propagation of new agents cause the movement of the wave.

### 3.1 Mutation of parasites

In the first set of experiments, we only allowed parasites to mutate and only considered agents that were initially distributed on two halves of the circle. In total, we conducted four computational experiments. The detailed parameters characterizing these experiments are presented in Table 1 and described below. The parameters that were constant are included in the caption of the Fig 1.

**Table 1:** The parameters used in the experiments analyzing the mutation of parasites that varied between experiments. *a*_*P*_0__ denotes the initial value for the affinity of parasites towards replicases, *l*_*P*_0__ denotes the initial value for the probability of a parasite being in a folded state, and *μ*_*a*_*P*__, *μ*_*l*_*P*__ denote probability of mutation of those two parameters, respectively, in each simulation step. When the parameter is immutable, the value of the mutation is not applicable.

Experiment	*a*_*P*_0__	*l*_*P*_0__	*μ*_*a*_*P*__	*μ*_*l*_*P*__
1	0.55	0.2	0.01	0.01
2	0.55	0.0–1.0, step 0.1	N/A	0.19
3	0.0–1.0, step 0.1	0.2	0.19	N/A
4	0.0–1.0, step 0.1	0.2	N/A	0.19

During the first experiment, we allowed the affinity of parasites towards replicases *a*_*P*_, and the probability of a parasite being in a folded state *l*_*P*_ mutate slightly. During the remaining experiments, the mutation probabilities *μ*_*a*_*P*__ and *μ*_*l*_*P*__ were increased to make the influence of mutations easier to observe. Moreover, to verify how one mutational probability influences the behavior of the system, only one of these parameters was mutating while the other was immutable. We varied the initial values of *a*_*P*_ (denoted as *a*_*P*_0__) or *l*_*P*_ (denoted as *l*_*P*_0__) from 0 to 1 to observe how various initial values influence the survivability of the system. In all experiments, the affinity of replicases towards replicases was equal to *a*_*R*_ = 0.7 and the replicases were catalytically active regardless of their folded state. The value of *a*_*R*_ differs from the value that was used in the experiments by Takeuchi and Hogeweg [33], where it was set to 0.6. We also examined the value *a*_*R*_ = 0.6; however, closer investigation showed that while a slightly higher value of this parameter equal to 0.7 does not change the characteristics of the system, it does give better results in terms of a more stable coexistence between parasites and replicases, meaning that we could more easily observe the emerging behaviors in the system. When *a*_*P*_0__ or *l*_*P*_0__ were immutable, their values were equal to 0.55 and 0.2, respectively. These values come from the ODE analysis for the parasite-replicase system made by Takeuchi and Hogeweg [33]. They allowed for the stable coexistence of the parasites and replicases modeled by the ODE assuming no mutation. They also allow the survival of both species in the CA experiments completed by Takeuchi and Hogeweg and worked correctly in our multi-agent model.

**Fig 3:**
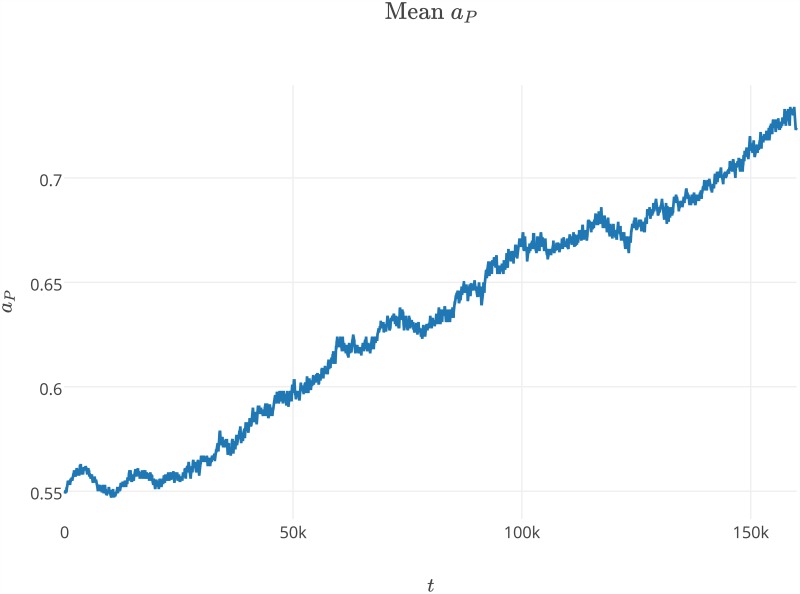
Average values of *a*_*P*_ changing over time during the first experiment. The values of parameters used during the experiment are presented in Table 1 and label of Fig 1.

**Fig 4:**
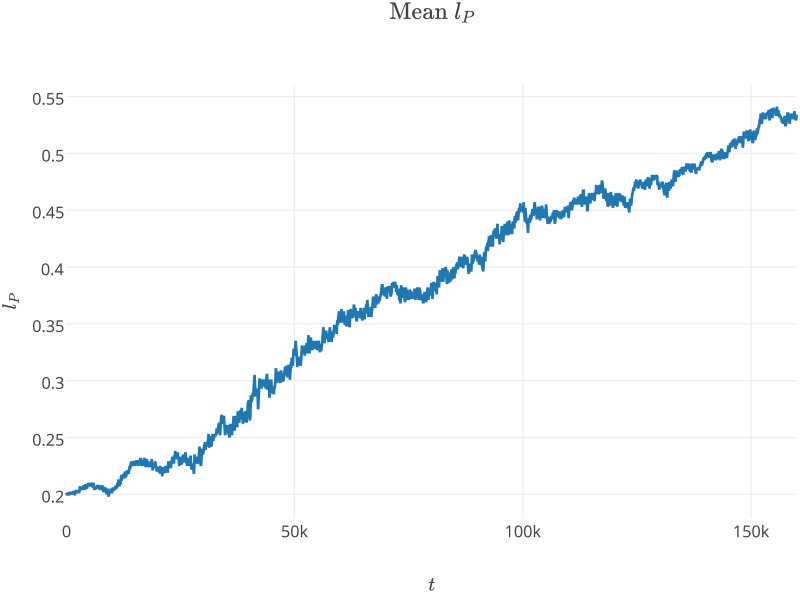
Average values of *l*_*P*_ changing over time during the first experiment. The values of parameters used during the experiment are presented in Table 1 and label of Fig 1.

The experiments we performed showed that the system was able to survive with a wide range of parameters values. The results show that our model, which incorporated space, was able to preserve the stable coexistence of the modeled agent species, despite the evolutionary instability of the replicase-parasite system [54]. In the first experiment, we observed that the average values of *a*_*P*_ and *l*_*P*_ steadily increased from the beginning of the simulation (Figs 3 and 4). We also observed that parasites increase their average affinity towards replicases *a*_*P*_ through evolution, but that increasing the average *l*_*P*_ malso means that parasites spend more time in a folded state (Figs 4 and 2). These two trends are in opposition to each other, as the former makes parasites more parasitic, the latter makes them less harmful. Increasing the time that parasites spend in a folded state appears to be unreasonable from the parasite point of view, as parasites in a folded state cannot be replicated and have no predefined function. This was also observed by Takeuchi and Hogeweg [33]. Their results regarding the surface model show that the observed trajectories of the *a*_*P*_ and *l*_*P*_ population averages can be separated into two phases, short-term and long-term evolution. In the first phase, the values of *a*_*P*_ and *l*_*P*_ are not changing. In the second phase, both observed values are increasing. In our experiment the first phase is very short and lasted approximately 25k steps.

**Fig 5:**
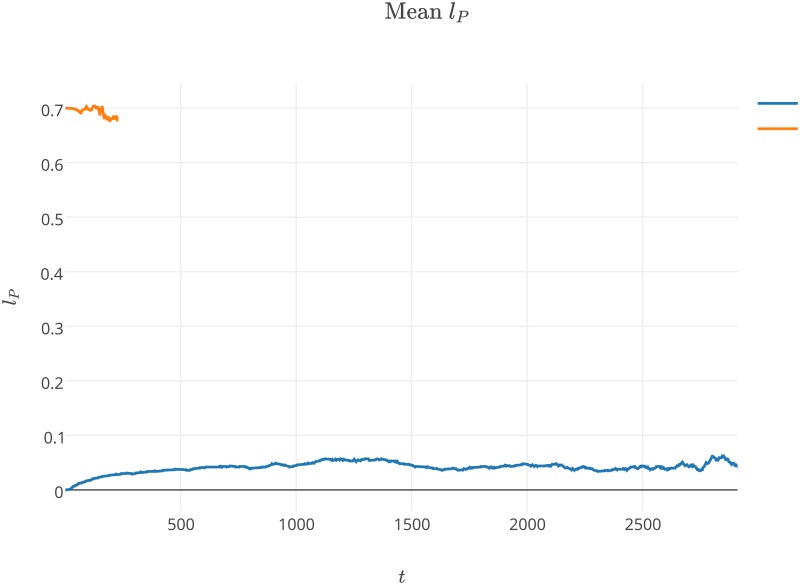
Average values for *l*_*P*_ during the second experiment with *l*_*P*0_ = 0.0 and *l*_*P*0_ = 0.7. The values of parameters used during the experiment are presented in Table 1 and label of Fig 1.

**Fig 6:**
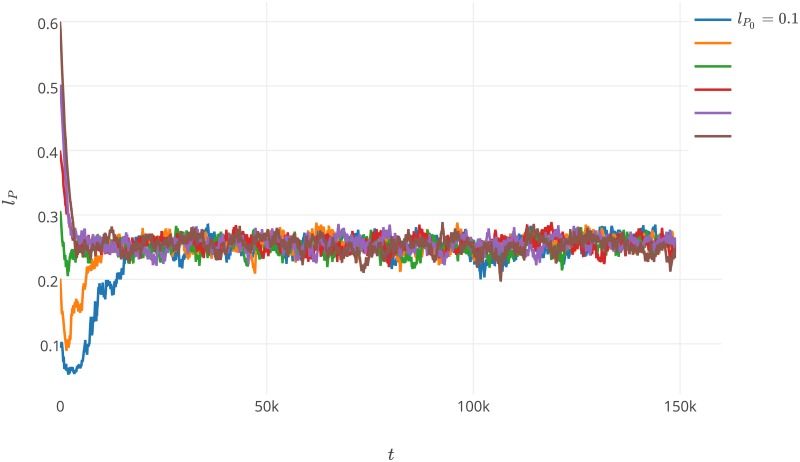
Average values for *l*_*P*_ during the second experiment with *l*_*P*0_ ranging from 0.1 to 0.6 (0.1 step). The values of parameters used during the experiment are presented in Table 1 and label of Fig 1.

After reproducing the Takeuchi and Hogeweg experiment, we performed an experiment where only *l*_*P*_ is mutable to better understand the obtained results. Moreover, we wanted to extend these results and further investigate how various mutable *l*_*P*_ initial values influence the behavior of the system, while the affinity towards replicases is immutable. Investigation of *l*_*P*_0__ varying from 0 by 0.1 to 1 showed that a stable coexistence is possible assuming quite low to average values for *l*_*P*_0__ (0.1 ≤ *l*_*P*_0__ ≤ 0.6). When *l*_*P*_0__ is extremely low (*l*_*P*_0__ = 0), parasites can easily form complexes with replicases and their virulence is so strong that they exploit the replicases so quickly that they kill their host, and as a consequence, they kill themselves. High *l*_*P*_0__ values (*l*_*P*_0__ ≥ 0.7) make parasites inaccessible to replicases as parasites spend most of their time in the folded state, and therefore do not have the chance to replicate and disappear. The average values for *l*_*P*_ during the initial settings for *l*_*P*_0__ = 0.0 and *l*_*P*_0__ = 0.7 are depicted in Fig 5. As presented in this figure, the system dies quickly. The results show that the system with mutable *l*_*P*_ is able to stabilize its behavior assuming that the values of *l*_*P*_0__ are in the range of 0.1 ≤ *l*_*P*_0__ ≤ 0.6. In such a case, the average value of *l*_*P*_ quickly stabilizes between 0.2 and 0.3 and stays in this range during the simulations (Fig 6). A closer look into the individual waves reveals that as the simulation progresses, the back side of a wave starts to be composed of parasites whose *l*_*P*_ value is continuously decreasing. Therefore, the parasites tend to maximize their chances of complex formation and become more parasitic. Takeuchi and Hogeweg provided an explanation for why the average value of *l*_*P*_ is maintained at a constant level for the whole system [33]. This is achieved due to the interplay between evolution at the level of waves and within waves. While the individual waves undergo an aging-like process caused by the maximization of the virulence of parasites forming a wave, this is counteracted by the high fecundity of waves composed of parasites that have relatively low complex formation abilities.

**Fig 7:**
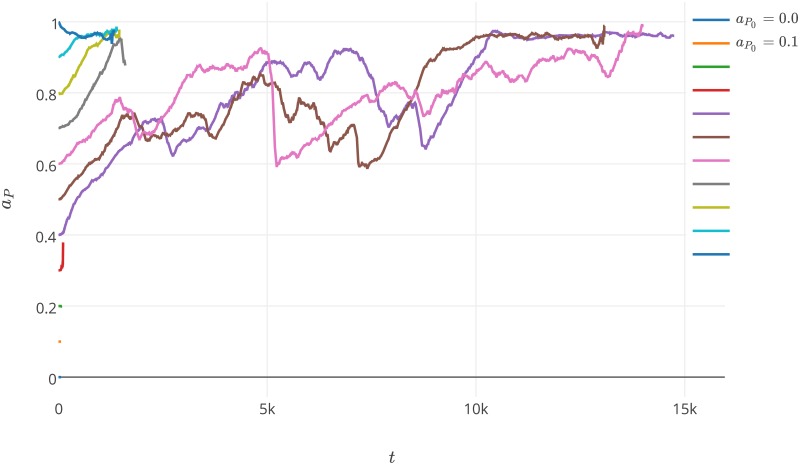
Average values for *a*_*P*_ during the third experiment with *a*_*P*0_ ranging from 0 to 1. The values of parameters used during the experiment are presented in Table 1 and label of Fig 1.

Another experiment verified how the initial value for the affinity towards replicases affects the behavior of the system. During this experiment, we varied the value of *a*_*P*_0__ and let it mutate, while *l*_*P*_ was immutable. The results showed that a stable coexistence was impossible in this case, regardless of the initial values for *a*_*P*_. The maximal number of steps for which system the preserves the investigated species is less than 16k (Fig 7). When *a*_*P*_0__ is low (*a*_*P*_0__ ≤ 0.3), parasites cannot easily form complexes with replicases, and as a consequence they quickly die. When *a*_*P*0_ is medium and high (0.4 ≤ *a*_*P*_0__ ≤ 0.9), parasites rapidly increase their average *a*_*P*_ value. In this case, the increase in the affinity towards replicases is not mitigated by an increase in the *l*_*P*_ values as was observed in the first experiment, and the system dies as a result system. For an extremely large value of *a*_*P*_0__ = 1, the increase in the *a*_*P*_ values cannot occur because it is already at a maximum from the beginning of the simulation. In fact, the system tends to minimize the threat of too high of an affinity by decreasing the *a*_*P*_ values, though this is not able to defy the parasites. As a consequence, when *a*_*P*_0__ > 0.4 the parasites exploit the hosts so strongly that the hosts cannot replicate themselves and the parasites kill the system.

**Fig 8:**
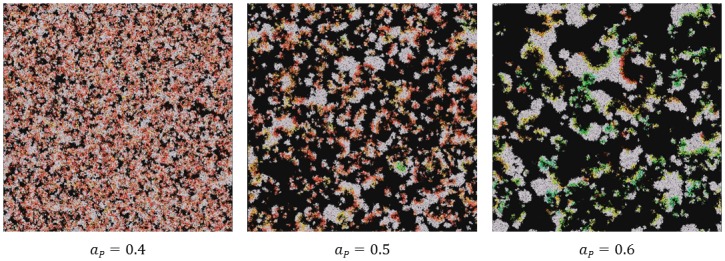
Screen shots from the fourth experiment for various of *a*_*P*_ initial values taken after approximately 80k simulation steps. The values of parameters used during the experiment are presented in Table 1 and label of Fig 1.

**Fig 9:**
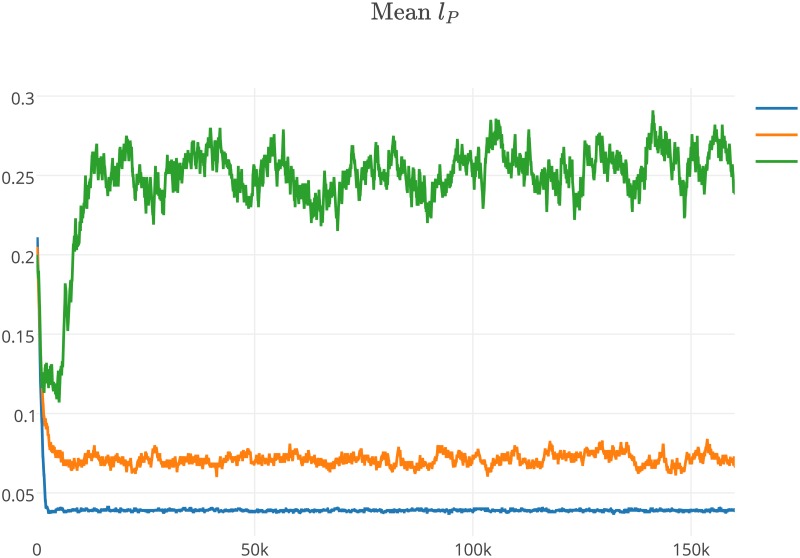
Average values of *l*_*P*_ during the fourth experiment when *a*_*P*_0__ = 0.4 (blue), *a*_*P*_0__ = 0.5 (orange) and *a*_*P*_0__ = 0.6 (green). The values of parameters used during the experiment are presented in Table 1 and label of Fig 1.

Taking into account previous experiments, we wanted to further investigate the role of the *a*_*P*_ parameter on system survival. Therefore, we conducted the fourth experiment in which we varied the initial value of *a*_*P*_0__ but did not allow it to mutate, instead *l*_*P*_ was mutable. The mutation of *l*_*P*_ is necessary to possibly counteract the initial affinity settings towards replicases and assure (at least theoretically) the stable coexistence of parasites and replicases. The results from the experiment show that when *a*_*P*_0__ is high (*a*_*P*_0__ ≥ 0.7) parasites bind much better than replicases and that they strongly exploit their host wave, kill it and lead to their own destruction. At low *a*_*P*_0__ values (*a*_*P*_0__ ≤ 0.3)), the parasites faced competition from the replicases that have higher average affinity values for replicases, were unable to make complexes as their affinity towards replicases was lower and disappear from the system. This situation seems to be unrealistic. We believe that loosing catalytic activity is easy, but that it is harder to completely loose affinity towards a given catalyst. Preserving catalytic activity often demands the presence of an accurate sequence and structure by the biomolecular catalyst, suggesting that it can easily disappear during replication via mutation. In contrast, motifs recognized by catalyst recognition motifs and binding RNA motifs are less conserved [55], suggesting that it is harder to loose affinity towards a catalyst, even given imperfect replication. Taking these data into account, when highly active replicases with average affinities towards replicases are subject to mutations during replication, they can easily become parasites; the affinity towards the catalyst does not have to change even if the catalytic activity is completely destroyed. Therefore, we expect that in the quasi-species population, in which there are replicases that have average affinity values towards themselves, parasites with similar average affinities towards replicases will form. When *a*_*P*_0__ is average (0.4 ≤ *a*_*P*_0__ ≤ 0.6), coexistence is possible. Moreover, within the range of average *a*_*P*_0__ values, these values affect the size of the waves. At *a*_*P*_0__ = 0.4 the waves are very small and not well-structured as all of them are connected (left image in Fig 8). At *a*_*P*_0__ = 0.5 the waves are bigger and better structured (middle image in Fig 8). The biggest and best structured waves can be observed when *a*_*P*_0__ = 0.6 (right image in Fig 8). Changes in the average *l*_*P*_ values shows the quick accommodation to a given affinity value and the stabilization of average *l*_*P*_ values approximately 0.05, 0.1 or 0.3 for *a*_*P*_0__ values equal to 0.4, 0.5 and 0.6, respectively (Fig 9).

Results obtained from the experiment 2–4 regarding the behavior of the system and observation of *l*_*P*_ values suggest that the evolution of *l*_*P*_ has a stabilizing role in the investigated system and that it is crucial for the coexistence of parasites and replicases.

### 3.2 Mutation of parasites and replicases

The previous section presented results from simplified experiments in which only parasites were the subject of changes. Here, we will consider the case in which mutations during replication affect not only parasites but also replicases. This should help to further investigate how the interplay between parasites and replicases affects the evolution of a system. To answer this question, we performed a fifth experiment where we let four parameters change during replication, the affinity of parasites and replicases towards replicases (*a*_*P*_ and *a*_*R*_, respectively) and the probability of either parasites or replicases (*l*_*P*_ and *l*_*R*_, respectively) being in a folded state. During the experiment, we performed several tests where we varied the initial *a*_*P*_ and *a*_*R*_ values to check how these changes affect the behavior of the system. The initial parameter values used during the experiment are shown in Table 2 with the information regarding whether the system survived or went extinct under these initial conditions. We analyzed the random and circular initial distribution of the agents. The initial values for *l*_*P*_ and *l*_*R*_ were always equal to 0.2. The probability of mutation at each replication event was set to 0.01 for each of the four mutation parameters. Similarly to the previous experiments, the parameters that are constant are included in the caption of the Fig 1.

**Table 2:** Initial values for the *a*_*P*_ and *a*_*R*_ parameters that were tested during the fifth experiment together with the information regarding whether the system survived after the specific initial distribution of the agents.

		Initial distribution of agents	
*a*_*P*_0__	*a*_*R*_0__	circle	random
0.3	0.5	extinct	extinct
0.4	0.6	**survive**	extinct
0.4	0.7	**survive**	extinct
0.55	0.6	extinct	extinct
0.55	0.7	**survive**	**survive**
0.8	0.6	extinct	extinct
0.8	0.7	extinct	extinct

**Fig 10:**
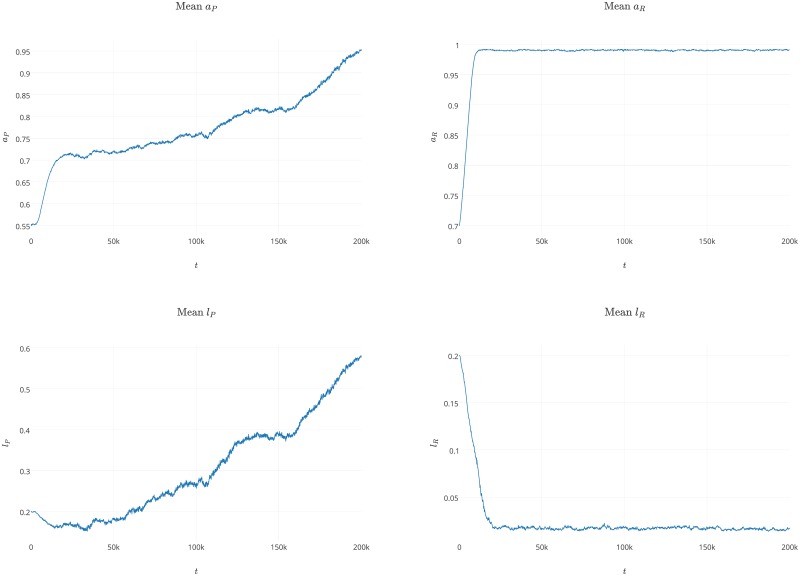
Plots showing the average *a*_*P*_, *a*_*R*_, *l*_*P*_ and *l*_*R*_ values during the simulations in the fifth experiment. The initial values for the investigated parameters were *a*_*P*0_ = 0.55, *a*_*R*0_ = 0.7, *l*_*P*0_ = 0.2, *l*_*R*0_ = 0.2. The probability of mutation for each parameter was set to 0.01 and the agents were initially distributed on a circle. The values of remaining parameters used during the experiment are presented in label of Fig 1.

For the random distribution of agents, the system was not able to survive under most of the tested conditions. For the nonrandom distribution, the system was able to maintain the stable coexistence of parasites and replicases and survive. This is possible assuming that the initial value of *a*_*P*_0__ is significantly lower than the initial *a*_*R*_0__ value and that *a*_*P*_0__ and *a*_*R*_0__ are high enough to assure the survival of their species. In the case of the systems that did not go extinct, the average *a*_*P*_ values were rapidly growing at the beginning of the simulation to 0.7, followed by a more smooth increase (Fig 10). The average *l*_*P*_ values dropped slightly at the beginning of the simulation and then slowly increased. This is the same trend that we observed during the first experiment and the same trend that was observed by Takuechi and Hogeweg [33] in the analogous experiment. The average *a*_*R*_ values grow very rapidly during the simulation to the maximum possible level close to 1. In turn, the average *l*_*R*_ value decreases very quickly and stabilizes at a very low level that is below 0.05. We observed that replicases increase their affinities towards replicases to the maximum possible value through evolution as well as decrease the time that they spend in a folded state. This means that replicases tend to be better recognized by other replicases and are favored as templates. This is due to a fact that replicases that spend more time in a folded state are replicated less often than replicases that are mostly in a template form.

## 4 Discussion

The parasite-replicase system model that is described in this paper explicitly considers parasites and replicases. It also explicitly treats the space similar to the previously analyzed CA model [33]), but accounts for more realistic diffusion based on the Brownian motions of molecules. The space in the model is two-dimensional, which is consistent with the assumption that placing RNA chains on a mineral surface can improve chain formation [10, 56–58]. Our model can be classified as a multilevel selection model of type 1 (MLS1), where the entities are the individual molecules rather than their groups. In contrast, a multilevel selection model of type 2 (MAS2) treats collectives as focal units [59], e.g., stochastic corrector model [28].

### 4.1 Stability of the replicase-parasite system

Our results show that even explicitly included parasites allow for a stable coexistence for both the replicase and parasite species, assuming that the parasites are mild.

From the evolutionary strategy point of view, we observed kin selection as it is known that in the case of strong altruists, they need assortative grouping to survive [60]. As replicases are challenged by parasites, the individual replicase is a strong altruist because it pays the absolute cost in fitness. The spatial consideration of the problem assures that replicases that are co-localized are often relatives. This is caused by finite diffusion; molecules diffuse slowly so newly created replicases with similar parameters are close to one another. Therefore, the limited dispersal of relatives allows for the maintenance of the strongly altruistic replicases. A more detailed consideration of our parasite-replicase multi-agent model, in which molecules are subject to mutation during replication, revealed that the affinity towards replicases is responsible for the structure of waves (Experiment 4), whereas the probability of being in a folded state seems to be the stabilizing factor in a system (Experiments 2–4). The interplay between replicases and parasites allows the system to survive (it has macroscopic stability) despite the microscopic (or local) instability caused by the parasites competing with the replicases. This is caused by the system dynamics, which are characterized by mesoscopic entities; in this case, the waves generated by the interactions between parasites and replicases. Similar results regarding multi-level selection were obtained by Takeuchi and Hogeweg [33]. In the cellular automaton model, these waves were called traveling waves [33]; however, due to the continuous space and more realistic diffusion implemented in our MAS approach, these traveling waves look more like an explosion of life.

Ma and Hu [61] performed a computer simulation that aimed to answer the question of whether cooperation between functional molecules (ribozymes) was possible during the early stages of evolution. They adapted a Monte-Carlo approach to design a grid Monte-Carlo model with a resolution at the level of individual molecules. The results demonstrated that not only is cooperation possible, but spread of different functional molecules can also occur, which are beneficial to different aspects of self-replication. Moreover, these molecules could survive despite the threat posed by parasites. What is more, the parasites became extinct during the simulation even if they act as better templates. The reason for the parasite extinction was not investigated in this paper. In our approach, we noticed completely different results, as the parasites were present in the simulated system even after a long run. Even if parasites were not placed *ad hoc*, parasites form due to imperfect replication, and it is hard to imagine that a replicator system could remove parasites.

### 4.2 Evolution of replication

Based on the experiment with evolving parasites and replicases, we see that even if the parasite-replicase system is stable and allows for the coexistence of these two quasispecies, the presence of a strong trade-off taking into account RNA folding favors the evolution of replicases in the form of a template rather than in a folded form. Replication is fastest on unfolded, short and linear templates, whereas catalytic activity requires that the polymer is folded into relatively compact and stable spatial structures, which often requires the existence of long chains. Thus, a good template is unlikely to be a good catalyst at the same time. Therefore, this might be an obstacle to further improvements and evolvability of efficient replicases. If RNA fulfilled both roles of storage of information and the main catalyst, evolution could have stuck, which obviously did not occur. This could be the reason why RNA stopped fulfilling the globally role of both the catalyst and template during the later stages of evolution. The division of function into a DNA template and protein catalysts with RNA being important intermediate molecules affected the vitality and behavior of the system, and allowed the evolution of better catalysts that ensured further development. This was also confirmed by the work of Takeuchi et al. [62]. They showed that after the introduction of DNA, RNA does not have to make a trade-off between being a catalyst and a template at the same time because it is unnecessary for RNA to serve as a template. Moreover, according to the paper, the lack of catalytic activity in DNA by itself can generate a sufficient selective advantage for RNA replicator systems to produce DNA.

Scheuring [63] theoretically analyzed the possibility of avoiding the error threshold in early replicator systems by analyzing non-enzymatic and enzymatic replication. For the case of enzymatic replication, he suggested that longer ribozymes could be more precise replicases and that they would consequently be fitter and might be maintained and spread in a quasispecies population. However, he did not consider that if longer molecules fold into complex shapes that give them better catalytic properties, their replication would be much more difficult. Therefore, longer molecules, even if they may be advantageous for the system, are not easy to maintain in the replicating population. For this reason, adaptive traits cannot be preserved if the same molecule has to serve as both a catalyst and a carrier of genetic information. Indeed, our results show that replicases tend to minimize the time they spend in a folded state and tend to maximize their availability as templates.

In turn, Szabó et al. [37] took a more detailed look into the ability of replicating RNA systems to evolve and maintain better replicases. They considered the following three parameters for replicating molecules: (1) replicase activity, (2) replicase fidelity and (3) template efficiency, all of which were based on the primary structure of the simulated molecules. This approach modeled the presence of different molecular domains responsible for different molecular traits. Moreover, these three replicase traits were expected to conflict with one another. Interestingly, it turned out that selection favored molecules bearing all three domains simultaneously during the simulation due to the reciprocal molecular altruism on a surface.

In the above model by Szabó et al., the modeling of separate domains that were responsible for specific functions allowed for an adaptive evolution of better replicases. In our multi-agent approach, the presence of a specific binding site for a replicase (template efficiency) is expressed as the affinity of a molecule towards replicases *a*. Moreover, in our model, the efficiency of a template is also affected by its availability for replicases in an unfolded state (expressed by *l*). Both mentioned parameters could potentially mutate during our simulations. In contrast, the replicase fidelity (expressed as a mutation probability) together with the replicase activity (expressed as a rate of replication) were maintained at a constant level during our simulations. Instead of this and according to Takeuchi and Hogeweg, we assumed that replication is limited by the requirement to form complexes rather than limiting itself directly. The complex formation depends on the values of *a* and *l*. In our case, none of the tracked properties were modeled as separate domains, instead they were attributed to the molecule as a whole. In the Szabó et al. model there were no trade-off between properties. This difference might be the reason why we observed only the selection of replicases towards better templates. These is because, better folded molecules, even if they could be potentially better replicases, cannot survive in this case because they will not be efficiently replicated.

Related work by Ma et al. [64] showed that primordial RNA replicases may have appeared in a nucleotide pool, spread and evolved into more efficient RNA replicases provided that they recognized their own sequences and their complements as catalytic targets. This assumption has been discussed by the authors who remarked that they are not sure how this condition could be fulfilled. They claimed that a plausible mechanism could be through Watson-Crick pairing similar to the recognition mechanism of group I ribozymes [65]. However, group II ribozymes lack phylogenetic conservation in ribozyme and substrate sequences near group II intron target sites. Instead, these ribozymes detect a structure at the junction between the single and double-stranded residues on the bound substrate [66] that is more robust than sequence recognition. In fact, many RNA motifs [67] do not have well-conserved primary sequences, e.g., RNA zip codes [55]. In the Ma et al. [64] work, the parasites did not pose a threat to the evolving system. When they adopted more plausible strategies by recognizing only part of a catalytic domain (as a motif), replicase emergence was hindered by the presence of parasites. Moreover, their results did not show that limited dispersal alone could ensure the emergence of primordial replicases and their evolution as in Szabó et al. [37]. Finally, even if the Ma et al. model was of micro-resolution and various factors had been taken into account, the chain folding problem was not investigated. Again, the lack of a trade-off imposed by incorporating the folding of RNA into the model was probably the reason why they did not observe the evolution of replicases towards being better templates.

In the light of our results, especially interesting is the work by Könnyü et al. [68]. They assumed that parasites can evolve into replicases. However, metabolically cooperating replicases (that is the replicases that enhance metabolism-producing monomers for replicase growth) which were also incorporated into their model, could not mutate based on the assumption that they are strictly constrained from changing by their spatial structure and monomer sequence due to the specific enzymatic function they have. In their model, non-enzymatic and enzymatic replication could occur. They showed that the coexistence of beneficial catalysts and parasites is possible and that selection favors RNAs that are good templates but not good catalysts or RNAs that are good templates and at the same time are effective replicases. These two possibilities came from the assumption that, similar to our approach, being a good template and having good replicase activity was a trade-off relationship. In this case, they directly influenced the strengths of the trade-off. Strong trade-offs selected the replication of efficient templates without catalytic properties, whereas weak ones selected RNAs that are both efficient templates and catalysts. In our research, this trade-off is always strong, which is why we observed only the selection of better templates. Whether this trade-off can be assumed to be strong or not in reality is an open question that probably requires more consideration.

Colizzi and Hogeweg [69] analyzed a system similar to ours, a minimal replicator system consisting of parasites and replicases. Both molecule types could change their complex formation rates via directionless mutation. Their results showed that if parasites are strong, replicators evolve high complex formation rates. In contrast, weak parasites do not lead to the evolution of higher association rates, and a system without parasites evolves towards minimized complex formation to a minimal viable value of 0.05. Our interpretation of results is different than Colizzi and Hogeweg’s as they considered only complex association rate of replicases as a whole, whereas we considered two important properties affecting complex association rate: affinity towards replicases and folding of RNA. In our model, replicases that spend more time in a folded state, have association rate smaller. Increase in the association rate of replicases is achieved by increasing the affinity of replicases towards other replicases and decreasing the time they spend in a folded form, therefore favoring them as templates. So when we consider the problem from a viewpoint of folding of RNA chain that affects association rate of replicases, it appear that dual role of replicases (catalysts and template) itself is something that could be an obstacle to further evolution.

Comparison to the related works [37, 63, 68, 69] shows how the basic assumptions embedded in a model can influence the results and their understanding. The number of degrees of freedom on which evolution acts can in fact influence the selection pressure. Modeling RNA properties as separate functional domains and taking into account the RNA chain folding problem are two fair assumptions. As future work, it would be interesting to incorporate both of these properties into one model and observe the results. Moreover, the investigation of how replication rate is affected by the selection would also be an interesting investigation. Our MAS approach is perfect for these purposes, as manipulating agent properties is straightforward.

### 4.3 The role of parasites

Because mutations are inevitable, especially during the early stages of the RNA World when the mechanism that corrects mismatched nucleotides had not been evolved yet, parasites were always present in the quasispecies. Moreover, as a loss of a catalytic activity is much easier than the loss of an affinity towards replicases, the once formed parasites were subsequently copied and further mutations could occur. In this way, parasites could freely roam through the sequence space [70] and, occasionally, new functions could be developed. Some mutations may have provided advantages to the system, and, if so, they could be further improved by evolution. For example, the mutant could become a new replicator, maybe even better than the previous one, or it could become a nucleotide synthase that produced building blocks for new chains. Moreover, the mutant could even evolve completely different functions and become for example an amphiphilic synthase, which produces protocell-membrane elements. The presence of amphiphilic elements in a neighborhood could allow for the direct compartmentalization of the replicator-parasite system and, by that, provide a way for one of the earliest major evolutionary transitions, the formation of protocells [71, 72]. Moreover, from the theoretical point of view, this could introduce completely different selection mechanisms (MLS2) into play in the evolution of life [28]. If it happened that the result of mutation was a trans-aminoacylator, which is possible as only 5 nucleotides are crucial for performing this function [73], then a route for the formation of a translation system could evolve. This makes our viewpoint feasible, because of the presence of a strong trade-off imposed by the double role of RNA replicases connected to their structure (folded versus unfolded state), the division of labor into a template capable of storing the information and a catalyst would be necessary for further evolution.

In this context, the article of Takeuchi and Hogeweg [74] shows that when the genotype-phenotype mapping of individual replicators is considered, the replicase—parasite interaction leads to the evolution of more lineages of parasites and replicases. Therefore parasites are not the threat to the system. They rather stimulate speciation and the evolution of more complex ecosystem.

## 5 Conclusions

During our experiments, we observed mesoscopic entities resembling traveling waves known from the Takeuchi and Hogeweg CA model. However, in our case, because of the more continuous treatment of the space, these mesoscopic entities looked more like explosions of life. We confirmed the general findings from Takeuchi and Hogeweg [33] regarding multilevel selection governing the evolution of the replicase-parasite surface model. Interplay between parasites and replicases makes the system stable, which is caused by evolution acting on the mesoscopic entity level and between them. However, even if stable coexistence is possible in a spatially extended system, this still does not mean that evolution leads to more efficient replicases. Instead, evolution tends to decrease the time replicases spend in a folded state, this way increasing their availability as templates. As replication is performed by RNA replicases that also store the necessary information for creating new instances of themselves, the existence of a trade-off that takes into account RNA folding could still pose a serious obstacle to the evolution. Thus, to evolve life as we know it, separation of the roles performed by replicases, information storage and replication of the information, into two molecules appears to be one of the crucial events that had to occur early in the stages leading to life.

## Supporting information

S1 Video Experiment 1, part 1. First part of the movie that was recorded during the simulation of the first experiment. The video was split equally to four parts because of file size constraints. The full movie in higher quality is also available on the YouTube (https://www.youtube.com/watch?v=mKpiUH0iDoQ). The values of parameters used during the experiment are presented in Table 1 and label of Fig 1. Several frames from the movie are also presented in Fig 2.
(MP4)

S2 Video Experiment 1, part 2. Second part of the movie that was recorded during the simulation of the first experiment. The video was split equally to four parts because of file size constraints. The full movie in higher quality is also available on the YouTube (https://www.youtube.com/watch?v=mKpiUH0iDoQ). The values of parameters used during the experiment are presented in Table 1 and label of Fig 1. Several frames from the movie are also presented in Fig 2.
(MP4)

S3 Video Experiment 1, part 3. Third part of the movie that was recorded during the simulation of the first experiment. The video was split equally to four parts because of file size constraints. The full movie in higher quality is also available on the YouTube (https://www.youtube.com/watch?v=mKpiUH0iDoQ). The values of parameters used during the experiment are presented in Table 1 and label of Fig 1. Several frames from the movie are also presented in Fig 2.
(MP4)

S4 Video Experiment 1, part 4. Fourth part of the movie that was recorded during the simulation of the first experiment. The video was split equally to four parts because of file size constraints. The full movie in higher quality is also available on the YouTube (https://www.youtube.com/watch?v=mKpiUH0iDoQ). The values of parameters used during the experiment are presented in Table 1 and label of Fig 1. Several frames from the movie are also presented in Fig 2.
(MP4)
